# Correlational analysis between salivary and blood glucose levels in individuals with and without diabetes mellitus: a cross-sectional study

**DOI:** 10.1080/00016357.2023.2267678

**Published:** 2024-03-26

**Authors:** Laasya Shettigar, Sanchita Sivaraman, Rohini Rao, Sanjana Akhila Arun, Aditi Chopra, Shobha U Kamath, Raju Rana

**Affiliations:** a Department of Periodontology, Manipal College of Dental Sciences, Manipal, Manipal Academy of Higher Education, Manipal, India; b UBC School of Population and Public Health, British Columbia, Vancouver, Canada; c Department of Data Science and Computer Applications, Manipal Institute of Technology (MIT), Manipal, Manipal Academy of Higher Education, Manipal, India; d Department of Biochemistry, Kasturba Medical College and Hospital, Manipal, Manipal Academy of Higher Education, Manipal, India

**Keywords:** Diabetes mellitus, type 2 diabetes mellitus, non-insulin-dependent diabetes mellitus, saliva, blood, blood glucose

## Abstract

**Objective:**

To estimate the association of patient-related demographic, socioeconomic status, physical activity, stress, and dietary factors influencing the relationship between salivary and blood glucose levels in individuals with and without diabetes mellitus (DM).

**Method:**

This cross-sectional study was conducted on 166 participants with and without DM. Saliva and blood were collected to estimate the glucose levels. Age, gender, occupation, socioeconomic and education level, BMI, hip to waist circumference, stress, dietary pattern, lifestyle, physical activity, family history of diabetes, and type of diabetes were recorded. The association of saliva to predict blood glucose levels was analysed using Spearman Rank Correlation and how these patient-related factors influence the correlation was estimated for future machine learning models. The difference in medians for various groups was calculated using the Mann-Whitney U Test or Kruskal Wallis Test.

**Results:**

Blood glucose level is not significantly correlated to salivary glucose level. However, a statistically significant difference in the median blood glucose levels for diabetic participants (median = 137) compared to healthy controls (*p*-value < .05) was noted. The correlation between blood and salivary glucose was more positive for higher levels of glucose (Spearman 0.4). Age, alcohol consumption, monthly wages, intake of vegetables, and socioeconomic status affect blood glucose levels.

**Conclusion:**

A correlation between saliva and blood glucose levels in healthy individuals was weak. Saliva should only be used as a monitoring tool rather than a diagnostic tool and is more reliable for patients with poorly controlled diabetes mellitus.

## GRAPHICAL ABSTRACT

**Figure:**
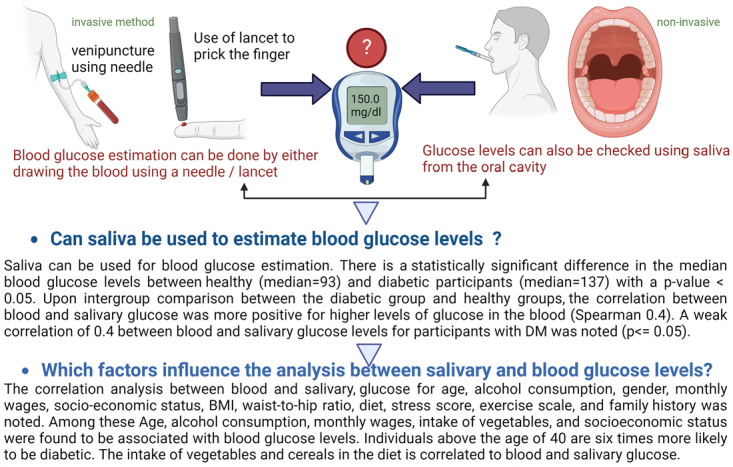

## Introduction

Diabetes mellitus (DM) is a group of metabolic diseases characterized by hyperglycaemia (increased blood glucose levels) either due to defects in insulin secretion, insulin action, insulin resistance, or both [1]. There are various forms of DM such as Type 1, Type 2, and gestational DM [2]. Type 2 DM is the most common form of DM. It occurs when cells do not respond to insulin (insulin resistance), and in turn, it reduces or inhibits the ingress of glucose into the cell. The glucose that is not utilized by the cells enters the blood circulation (free glucose) and results in hyperglycaemia. An individual with a fasting blood glucose level of more than 110 mg/dl or a glycated haemoglobin test (HbA1c > 6.5) is confirmed to have DM [3–4].

Around 463 million people between the age of 20–70 years are estimated to be living with DM, which represents 9.3% of the total world population. It is predicted that by 2030, 366 million individuals will suffer from DM, out of which 90% will suffer from Type 2 DM [5]. It is also estimated that 50.1% are undiagnosed cases and most of the population that are undiagnosed with DM (84.3%) belongs to low and middle-income countries [5]. The low diagnostic rate of DM is attributed to the high cost of testing DM, poor access to hematological laboratories, financial constraints, poor compliance and attitude of the patient towards regular health check-ups, especially in underdeveloped rural and sub-urban areas. The fear of needle pricks and withdrawing blood in a vacutainer using needles and syringes also preclude many from regular hematological testing [6].

To overcome the fear of needle pricking, currently hematological analysis for DM can be done either by using a lancet and glucometer [7]. Glucometers and the use of glucometer strips are less invasive and quicker methods of blood glucose estimation. However, the fear of needle prick and withdrawal of blood still exists among individuals using a glucometer, as it creates a sense of anxiety and stress among many individuals [8–11]. Studies have found that many individuals fear ‘self-testing’ at home and around 57% of individuals fear needle pricks even by a lancet [12–16]. Additionally, the armamentarium of using a glucometer is expensive for poor patients and some patients do not want to prick themselves regularly. Thus, glucometers are used mainly by people from the middle-higher socio-economic state, those with higher educational qualifications, and those who know how to use them [11]. A study by Farhan et al. (2017) found that the prevalence of home glucometer usage was only 59%. The cost of using a glucometer, refill lancets and strips are major factors limiting its use. The study also found that high socioeconomic status (*p* < .001), receiving care from private institutions (*p* < .001), higher education (*p* < .001), a family history of diabetes (*p* = .001), awareness regarding diabetes (*p* < .001), having diabetes for > five years (*p* < .001), and managing diabetes *via* pharmacological interventions (*p* < .001) (versus diet and exercise) were positive predictors of glucometer usage [14–16]. Additionally, the use of needles and lancets can be dangerous for individual with a bleeding disorders, those on anticoagulants and antiplatelet medications; as the pricked site may take a long time to clot [17]. Thus, the need for developing a viable and less invasive prick-free method for glucose estimation is always felt and researched [12–16].

Saliva has been tested as a potential biological fluid for estimating blood glucose levels [15–26]. Many studies have tested and compared the potential of using saliva compared to blood for glucose estimation and reported both positive and negative results [3,8,17–30]. A systematic review by Mascarenhas et al. (2014) evaluated the effectiveness of salivary glucose in estimating glycaemia and HbA1c and found that there is a large positive effect of Type 2 DM over salivary glucose (Hedge’s *g* = 1.37). The overall global correlation coefficient (r) between salivary glucose and glycaemia was large (*r* = 0.49). The strength of the correlation increased for higher glycaemia/HbA1c values [31–33]. Additionally, many studies have shown that the use of saliva for estimating blood glucose levels is influenced by many patient-related and environmental factors. Panchbhai et al. (2012) in their study concluded that ‘several factors such as age, stress, anxiety, diet, medical and drug history, smoking history, and nature of diabetes influence the salivary glucose levels and eventually its correlation to blood glucose level. Unless these factors are noted, salivary glucose cannot be employed to estimate blood glucose levels [19]. Although limited studies have previously discussed the role of these factors and how they affect the salivary and blood glucose levels, it is not clear how these factors would influence overall correlation if one has to utilize saliva for glucose estimation. To our knowledge, previous studies have not evaluated the correlation of these factors and how they influence the correlation between blood and salivary glucose levels. This evidence is vital for understanding how salivary glucose levels will change for a given individual and how one should use a multifactorial approach to estimate blood glucose levels using saliva more accurately. This is important as it would help data scientists, machine learning model developers, and clinicians before designing any app or algorithm that would utilize saliva for analyzing blood glucose levels. With this background, the study aims to estimate the association of various patient-related demographic, socioeconomic, stress, and dietary factors influencing the relationship between salivary and blood glucose levels in systematically healthy patients and patients with DM.

## Methodology

The study is designed as a cross-sectional correlational study conducted from December 2020 to July 2021 and was conducted following the STROBE and SAGER guidelines. The study was conducted at the Department of Periodontology, Manipal College of Dental Sciences, Manipal, in collaboration with the Clinical Haematology Laboratory, Kasturba Medical Hospital, Manipal. The study was conducted in accordance with the tenets of the Helsinki Declaration (as revised in 2013) and has been approved by the Institutional Review Committee of Kasturba Medical College and Kasturba Hospital (IEC no:443/2020). This study was also registered at the clinical trial registry, in India with registry no (CTRI/2020/ 12/029832). The informed consent was obtained from all study participants in a written and verbal manner explaining the methodology and rationale of the study.

### Sample size calculation

The total sample size was calculated considering Alpha, with Significance as .05, and Beta, with the power of the study at 80% (1-Power = 0.20); the standard deviation between the outcomes: 0.1 using the following formula was found to be 83 in each group.


$$\text{N}={\left(\frac{z_{\alpha }+z_{\beta }}{\left(0.5*\text{ln}\left(\frac{1+r}{1-r}\right)\right)}\right)}^{2}+3$$

### Inclusion and exclusion criteria

All individuals coming to the hematological laboratory at Kasturba Medical Hospital and the out-patient Department of Periodontology, Manipal were screened for the following inclusion and exclusion criteria:

Inclusion criteria:

1. All systemically healthy participants and participants diagnosed with controlled and uncontrolled Type 2 DM in the age range of 20–75 years who have come for blood glucose estimation were included.

**Exclusion criteria:**

1. Participants with any other systemic diseases other than DM.
2. Participants taking any medication or drugs other than anti-diabetic medications.
3. Participants taking any antibiotics or analgesics in the past month.
4. Participants having any active infection that requires emergency treatment at the time of recruitment.
5. Pregnant and lactating women to rule out gestational DM.
6. Mentally and physically challenged individuals.
7. Participants undergoing any radiation and chemotherapy.

After screening all the participants for the presence of DM and for healthy (non-diabetic individuals), oral and written informed consent was taken from all the participants. The following personal and demographic data was then collected on a printed data collection form by two investigators (LS and AC): age (in years), gender, address (district/place), occupation; gross income (in INR); no. of working hours; socioeconomic status (low/middle/high), education level; type of diabetes; family history of diabetes (father/mother/paternal/maternal grandmother/paternal/maternal grandmother); nature of diabetic medication and duration of DM. After recording the demographics, weight (in kg), height (in cm), hip circumference (in cm), and waist circumference (in cm) were recorded with a duly calibrated measuring tape. All participants were questioned about their lifestyle and levels of physical activity by using the International Physical Activity Questionnaires (IPAQ) index as described previously [34]. Based on the IPAQ, domain-specific scores for each type of physical activity were noted, and based on this the type of activities performed by each participant was divided into sedentary/low/moderate/ high activity. The participant’s diet history was recorded to note the type of diet, and the number of times the participant consumed fruits and vegetables (fibers), tanned food, hydrogenated and processed food, and alcohol. A standard diabetic and diet questionnaire as described previously was followed to note if the individual follows a healthy diet [35].

The individuals were also questioned regarding their stress levels by utilizing the Perceived Stress Scale (PSS)-10 [36]. A total score ranging from 0 to 40 was computed by reverse scoring the four positively worded items and then summing all the scale items. Higher scores indicated greater levels of perceived stress. Subscale scores were computed by summing the six negatively worded items (Items 1, 2, 3, 6, 9, and 10) for Factor 1 (‘Negative’) and the four positively worded items (Items 4, 5, 7, and 8) for Factor 2 (‘Positive’), with higher scores indicating greater negative distress/stress feelings and greater positive stress feelings and coping abilities, respectively. Following the collection of demographic data, physical activity score, diet chart, and stress score, all participants were requested to provide blood and salivary samples. The saliva and blood samples were collected by a trained technician as follows.

### Collection of saliva for salivary glucose estimation

The unstimulated whole saliva was collected using the ‘spitting method’ [37]. The unstimulated saliva was preferred to stimulated saliva as alterations in the salivary composition have been noted with stimulated saliva. All participants were requested to sit comfortably in an upright position. Participants wearing any denture or removable prosthesis wearers were asked to remove the prosthesis before the saliva collection. The subjects were requested to spit out the saliva accumulated in the mouth into an Eppendorf vial. 2–3 ml of unstimulated saliva were collected and stored at minus 80 degrees Celsius until further analysis. The saliva samples were first centrifuged at 3000 rotations per minute for 20 min to obtain a clear supernatant. The supernatant was used for the estimation of salivary glucose using the glucose oxidase end-point assay as described previously [37].

### Estimation of the blood glucose levels

2 ml of blood was collected. The patient was made to comfortably sit on a chair with arms extended straight from the shoulders onto a wooden plank. The antecubital fossa was exposed and a tourniquet was tied at 1.5–2 inches above the fossa. The area was cleaned and made sterile by using cotton wool soaked in methylated spirit. Using a BD vacutainer needle, the antecubital vein was punctured and 2 ml of whole blood was drawn. The hemostasis was achieved at the puncture site by pressing with a cotton plug soaked with spirit following which a bandage was applied. The blood was then centrifuged at 3000 rpm for about 5 min to separate the serum and evaluate the glucose level. One millilitre of glucose reagent was added to 10: l of the test sample and glucose standard. Both were incubated at 37 °C for about 10 min. The absorbance values were measured with a semi-automated analyzer [37–40].

### Statistical analysis

After data collection, all the details were compiled into a Microsoft Excel (version. 2019) The programming language used for performing the statistical analysis is Python 3.8.16. The packages used for analysis are Pandas (Version 1.3.5) and Scipy (Version 1.7.3). The packages used for visualizing results are Seaborn (Version 0.11.2) and Matplotlib (Version 3.2.2). Kolmogorov-Smirnov test and Shapiro–Wilk test for various significance levels were performed and the blood and salivary glucose features were not following a normal distribution. Since Pearson correlation assumes normality in data, we analyzed results using the Spearman Rank Correlation and checked its statistical significance. The median is chosen as the measure of centrality, and the minimum and the maximum of all variables were calculated and used to describe the distribution. The Student’s T-test and Unadjusted Odds Ratio were calculated for further understanding of the underlying relationships. The difference in medians for various groups was calculated using the Mann-Whitney *U* Test or Kruskal Wallis Test. The results are then tabulated and visualized as follows.

## Results

A total of 2,000 participants were screened and questioned to check the presence of Type 2 DM. Out of 2000, 166 participants (both healthy and diabetic participants) were recruited, of which 99 were females and 67 were males. The mean age ± SD for the females (*n* = 99) was 37.89 ± 18.52 and for males (*n* = 67) was 46.84 ± 17.673. The overall mean and standard deviation of age, among 166 samples were 41.5 ± .18.6; the Median [Min-Max] is 40 [20–78]. Based on the blood glucose level results, the participants were divided into the diabetic group and the healthy control. The results showed that there were 83 healthy controls (group A) (male:31; female:52) and 83 participants with DM (Group B) (male: 36; female:47). The mean age in the healthy control and diabetic individuals were respectively 33 and 49. The age and male-to-female distribution has been depicted in Table 1.

**Table 1:** Age and gender distribution of participants.

Groups	Gender	Gender-wise distribution (N)	Mean Age	Std Deviation	Min age	Q1	Median Age	Q3	Max Age
Group A (Healthy)	Female	52	30	14.4	20	22	24	27.75	74
	Male	31	40	18.19	20	24	31	54.5	74
Group B (Diabetic)	Female	47	46	19.04	20	24	52	62.5	76
	Male	36	53	14.73	20	47.75	54	63.25	78

**Figure 1:**
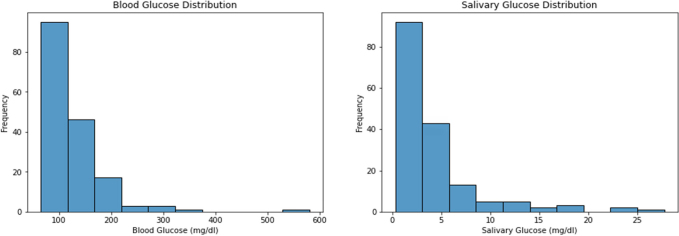
Describes the overall distribution of salivary and blood glucose levels.

Taking the overall cohort of 166 participants, the mean blood glucose was found to be 127.34 ± 58.2, and the median [Min-Max] was found to be 110.5 [65–581]). The mean and standard deviation for salivary glucose distribution was 3.94 ± 4.59 with a median [Min-Max] 2.5 [0.317–27.759] **(**Figure 1**)**.

**Figure 2:**
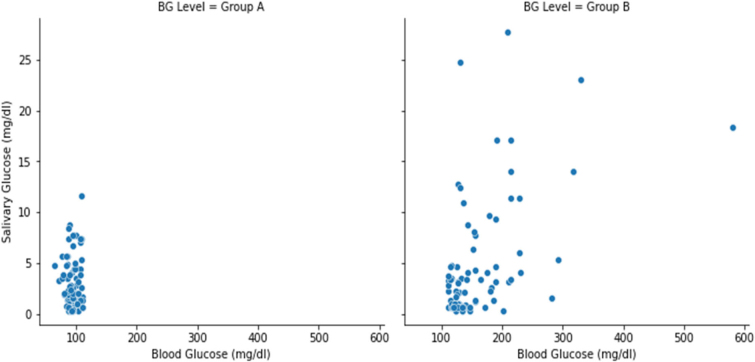
Schematic representation of the relationship between salivary and blood glucose level.

As per Spearman rank correlation (0.15), we found no correlation between blood glucose and salivary glucose overall, however, a statistically significant difference was noted between salivary and blood glucose levels for those with high blood glucose levels. Using the Spearman correlation, we noted a weak correlation of 0.4 between the blood glucose and salivary glucose levels for Group B (Diabetic) considering the significance level of p< = .05. Upon intergroup comparison between the blood and salivary glucose levels using the Mann-Whitney *U* tests between group A and group B, a statistically significant difference in the distribution of

blood glucose levels between group A and group B (*p*-value< .05) was noted, but the same could not be represented in the salivary glucose levels between group A and group B. **(**Table 2 **and** Figure 2**)**

**Table 2:** Group-wise comparison of blood and salivary glucose levels.

Groups	Blood Glucose Level Median [Min-Max] Mg/dl	Salivary Glucose Level Median [Min-Max] mg/dl	Group-wise correlation analysis of blood vs. salivary glucose
**Overall (*N* = 166)**	110.5 [65-581]	2.5 [0.317–27.759]	–
**Group A (Healthy) (*N* = 83)**	**93** [65-110]	2.42 [0.317–11.57]	**Spearman: (-0.02)** Pearson: 0.037
**Group B (Diabetic) (*N* = 83)**	**137** [111-581]	3.01[0.317–27.759]	**Spearman: 0.4** Pearson: 0.51

### Analysis between blood and salivary glucose with the demographic, socioeconomic, and diet data

The correlation analysis between blood and salivary glucose for age, alcohol consumption, gender, monthly wages, socio-economic status, BMI, waist-to-hip ratio, diet, stress score, exercise scale, and family history was noted. The results based on the Chi-square test showed an association of the following sociodemographic variables with blood glucose levels. Age (*p*-value = .00), alcohol consumption (*p*-value= .016), monthly wages (*p*-value = .009); socioeconomic status (*p*-value = .019) were positively correlated with the diabetic group with a statistically significant *p*-value < .05. The unadjusted odds ratio was computed and their statistical significance was tested against the confidence intervals. The statistically significant variables for blood glucose are as follows: The unadjusted odd ratio showed the well-known pattern that persons above the age of 40 are six times more likely to be diabetic. Participants with low monthly wages (<10,000 INR) are four times more likely to be diabetic. Participants from low socioeconomic status are three times more likely to be diabetic (Table 3).

**Table 3:** Association of sociodemographic variables with blood glucose (group a vs. Group B).

Variable	Variable Category	Blood Glucose GROUP B *n* = 83 (*n*%)	Blood Glucose GROUP A *n* = 83 (*n*%)	Chi-square p-value	Unadjusted Odds Ratio (Confidence Interval)
Age	Less Risk (< =40)	24 (28.92%)	60 (72.29%)	0.000	0.156 (0.079–0.306)
	More Risk (>40)	59 (71.08%)	23 (27.71%)		6.413 (3.264–12.601)
Alcohol Consumption	No	58 (69.88%)	44 (53.01%)	0.016	2.337 (1.216–4.491)
	Yes	22 (26.51%)	39 (46.99%)		0.428 (0.222–0.822)
Gender	Male	36 (43.37%)	31 (37.35%)	0.527	1.285 (0.690-2.392)
	Female	47 (56.63%)	52 (62.65%)		0.778 (0.418–1.449)
Monthly Wages	High	1 (1.20%)	3 (3.61%)	0.009	0.342 (0.035–3.359)
	Low	19 (22.89%)	6 (7.23%)		4.079 (1.533–10.856)
	Middle	58 (69.88%)	73 (87.95%)		0.358 (0.151–0.844)
Socio economic status	High	3 (3.61%)	4 (4.82%)	0.019	0.741 **(**0.161–3.417)
	Low	19 (22.89%)	6 (7.23%)		3.809 (1.436–10.109)
	Middle	61 (73.49%)	73 (87.95%)		0.379 (0.167–0.863)
BMI	Normal	43 (51.81%)	37 (44.58%)	0.535	1.336 (0.726–2.461)
	Obese	10 (12.05%)	10 (12.05%)		1.0 (0.392–2.546)
	Overweight	26 (31.33%)	34 (40.96%)		0.657 (0.347–1.243)
	Underweight	4 (4.82%)	2 (2.41%)		2.051 (0.365–11.515)
Waist-to-hip Ratio	High	26 (31.33%)	36 (43.37%)	0.243	0.596 (0.315–1.124)
	Low	46 (55.42%)	37 (43.37%)		1.623 (0.79–2.996)
	Moderate	11 (13.25%)	11 (13.25%)		1.000 (0.408–2.453)
Diet	Mixed	61 (73.49%)	62 (74.69%)	0.999	0.984 (0.488–1.982)
	Non-Veg	1 (1.20%)	1 (1.20%)		1.012 (0.062–16.462)
	Veg	20 (24.09%)	20 (24.09%)		1.0161 (0.499–2.071)
Stress Score	High	9 (10.84%)	13 (15.66%)	0.646	0.655 **(**0.263–1.628)
	Low	17 (20.48%)	17 (20.48%)		1.000 (0.471–2.125)
	Moderate	57 (68.67%)	53 (63.86%)		1.241 (0.651–2.365)
Exercise Scale	High	5 (6.02%)	8 (9.64%)	0.119	0.601 (0.188–1.919)
	Low	40 (48.19%)	30 (36.14%)		1.643 (0.883–3.059)
	Medium	9 (10.84%)	19 (22.89%)		0.409 (0.173–0.969)
	Sedentary	29 (34.94%)	26 (31.33%)		1.177 (0.616–2.249)
Family History	Father	20 (24.09%)	14 (16.87%)	0.335	1.565 (0.729–3.358)
	Mat grandfather only	1 (1.20%)	3 (3.61%)		0.325 (0.033–3.192)
	Mat grandmother only	1 (1.20%)	6 (7.23%)		0.157 (0.018–1.329)
	Mother	15 (18.07%)	13 (15.66%)		1.188 (0.526–2.681)
	No history	38 (45.78%)	39 (46.99%)		0.953 (0.518–1.754)
	Pat grandfather only	2 (2.41%)	4 (4.82%)		0.488 (0.087–2.738)
	Pat grandmother only	6 (7.23%)	4 (4.82%)		1.539 (0.418–5.667)

The assessment of the socio-demographic variables with salivary glucose some differences the in the median for some variables, such as diet, exercise, stress, and family history, the results were not statistically significant **(**Table 4**)**.

**Table 4:** Association of socio-demographic variables with the salivary glucose levels for both diabetic and non-diabetic.

Variable	Variable Category	Salivary Glucose Median [Min-Max]	Kruskal Wallis test (*p*-value)
Age	Less Risk (< =40) 84 (50.6%)	2.860 [0.317–12.709]	0.163
	More Risk (>40) 82 (49.4%)	2.006 [0.334–27.759]	
Alcohol Consumption	No 102 (61.45%)	2.54 [0.317–27.759]	0.943
	Yes 61 (36.75%)	2.46 [0.317–17.056]	
Gender	Male 67(40.36%)	2.675 [0.317–17.056]	0.700
	Female 99(59.64%)	2.460 [0.317–27.759]	
Monthly Wages	High 4(2.41%)	2.70 [0.635–3.490]	0.848
	Low 25(15.06%)	2.54 [0.334–27.759]	
	Middle 131(71.92%)	2.46 [0.317–24.749]	
Socio economic status	High 7(4.22%)	2.54 [0.600–4.320]	0.545
	Low 25(15.06%)	2.54 [0.334–27.759]	
	Middle 134(80.72%)	2.46 [0.317–24.749]	
BMI	Normal 80(48.19%)	2.122 [0.317–27.759]	0.559
	Obese 20(12.05%)	2.700 [0.600–17.056]	
	Overweight 60(36.14%)	2.54 [0.334–24.749]	
	Underweight 6(3.61%)	3.102 [0.317–4.760]	
Waist to hip Ratio	High 62(37.35%)	2.240 [0.317–27.759]	0.534
	Low 82(49.4%)	2.860 [0.317–24.749]	
	Moderate 22(13.25%)	2.441 [0.317–9.698]	
Diet Score	Mixed 123(74.1%)	2.540 [0.317–27.759]	0.389
	Non-Veg 2(1.2%)	1.136 [0.600–1.672]	
	Veg 40(24.1%)	3.235 (0.317–24.749)	
Stress Score	High 22(13.25%)	3.572 (0.334–24.749)	0.439
	Low 34(20.48%)	1.786 (0.317–12.374)	
	Moderate 110(66.27%)	2.54 (0.317–27.759)	
Exercise	High 13(7.83%)	2.220 (0.334–11.570)	0.496
	Low 70(42.17%)	3.085 (0.317–27.759)	
	Medium 28(16.87%)	2.50 (0.317–23.070)	
	Sedentary 55(33.13%)	1.900 (0.317–18.300)	
Family History	Father 34(20.48%)	2.26675 (0.317–18.30)	0.334
	Mat grandfather only 4(2.41%)	0.79250 (0.635–2.020)	
	Mat grandmother only 7(4.22%)	3.490 (0.600–8.770)	
	Mother 28(16.87%)	3.252 (0.334–24.749)	
	No history 77(46.39%)	2.260 (0.317–27.759)	
	Pat grandfather only 6(3.61%)	3.50 (0.317–7.01)	
	Pat grandmother only 10(6.02%)	3.27650 (0.432–12.709)	

**Figure 3:**
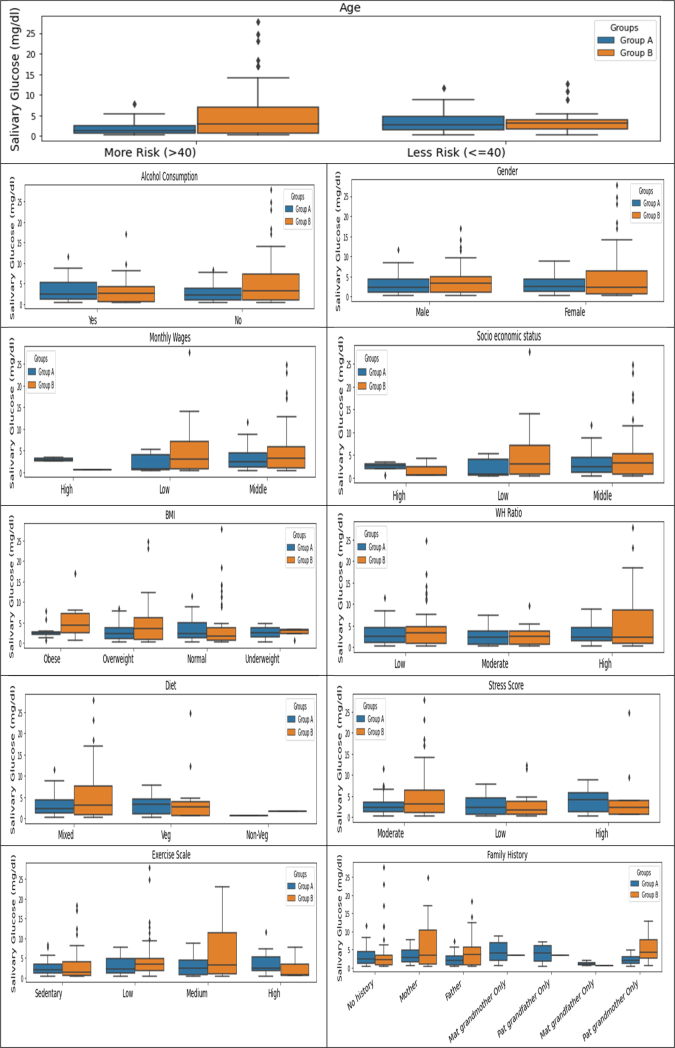
Group-wise analysis of socio-demographic variables with salivary glucose.

The intergroup comparison by the Mann-Whitney *U* test between the factors that affect the correlation between salivary and blood glucose levels for both groups A and group B showed that the age of the participants is the most significant factor influencing this correlation. For participants who were above 40 years of age, a statistically significant (with large effect) difference in the median between the salivary glucose levels for non-diabetic (median= 1.337) vs. participants diabetic (median= 3.010) was noted (Table 5 **and** Figure 3**)**. A positive correlation between blood glucose levels and consumption of fruits and vegetables was noted **(**Supplementary Table 1**)**. Participants who consumed vegetables (fresh, tinned, or frozen) and pulses like lentils, and kidney beans influenced the correlation between salivary and blood glucose levels. Stress in an individual is also associated with salivary and blood glucose levels **(**Supplementary Table 2**)**

**Table 5:** Analysis of socio-demographic variables with blood and salivary glucose between group A: (healthy) and group B: (diabetic).

Variable	Variable Category	Blood Glucose Level	Salivary Glucose Median [Min–Max]	Mann-Whitney U Test Statistic (*p*-value)	Effect Size For Mann-Whitney *U* Test
Age	Less Risk (< =40)	Group A 60 (71.43%)	2.700 (0.317–11.570)	730.5 (.921)	1.966
		Group B 24 (28.37%)	3.085 (0.334–12.709)		
	More Risk (>40)	Group A 23 (23.05%)	1.337 (0.334–7.720)	488.0 (.0496)	0.532
		Group B 59 (71.95%)	3.010 (0.334–27.759)		
Alcohol Consumption	Yes	Group A 39 (63.93%)	2.460 (0.317–11.570)	447.5 (.787)	1.464
		Group B 22 (36.07%)	2.635 (0.317–17.056)		
	No	Group A 44 (43.14%)	2.2805 (0.317–8.361)	1078.0 (.182)	0.795
		Group B 58 (56.86%)	3.240 (0.334–27.759)		
Gender	Male	Group A 31 (46.27%)	2.341 (0.317–11.570)	495.0 (.432)	0.987
		Group B 36 (53.73%)	3.339 (0.334–17.056)		
	Female	Group A 52 (52.53%)	2.460 (0.317–8.770)	1199.0 (.875)	0.859
		Group B 47 (47.47%)	2.260 (0.317–27.759)		
Monthly Wages	High	Group A 3 (75%)	2.860 (2.540–3.490)	3.0 (.5)	2.012
		Group B 1 (25%)	0.635 (0.635–0.635)		
	Low	Group A 6 (24%)	0.732 (0.334–5.351)	37.5 (.226)	0.515
		Group B 19 (76%)	3.010 (0.334–27.759)		
	Middle	Group A 73 (55.73%)	2.420 (0.317–11.570)	1952.0 (.446)	1.16
		Group B 58 (44.27%)	3.235 (0.317–24.749)		
Socio economic status	High	Group A 4(57.14%)	2.700 (0.600–3.490)	6.0 (1.0)	1.336
		Group B 3 (42.86%)	0.635 (0.635–4.320)		
	Low	Group A 6 (24%)	0.732 (0.334–5.351)	37.5 (.226)	0.515
		Group B 19 (76%)	3.010 (0.334–27.759)		
	Middle	Group A 73 (54.48%)	2.420 (0.317–11.570)	2122.0 (.642)	1.083
		Group B 61 (45.52%)	3.160 (0.317–24.749)		
BMI	Normal	Group A 37 (46.25%)	2.341 (0.317–11.570)	874.0 (.451)	0.674
		Group B 43 (53.75%)	1.672 (0.317–27.759)		
	Obese	Group A 10 (50%)	2.500 (0.600–7.720)	31.0 (.162)	1.251
		Group B 10 (50%)	4.342 (0.635–17.056)		
	Overweight	Group A 34 (56.67%)	2.320 (0.334–8.361)	353.5 (.189)	1.316
		Group B 26 (43.33%)	3.584 (0.334–24.749)		
	Underweight	Group A 2 (33.33%)	2.539 (0.317–4.760)	4.0 (1.0)	0.567
		Group B 4 (66.67%)	3.102 (0.668–3.344)		
Waist Hip Ratio	High	Group A 36 (58.06%)	2.340 (0.334–8.770)	459.0 (.903)	1.223
		Group B 26 (41.94%)	2.240 (0.317–27.759)		
	Low	Group A 36 (43.9%)	2.480 (0.317–11.570)	739.5 (.411)	0.779
		Group B 46 (56.1%)	3.252 (0.334–24.749)		
	Moderate	Group A 11 (50%)	2.341 (0.317–7.360)	58.5 (.921)	0.952
		Group B 11 (50%)	2.540 (0.317–9.698)		
Diet	Mixed	Group A 62 (50.41%)	2.381 (0.317–11.570)	1682.5 (.293)	0.96
		Group B 61 (49.59%)	3.170 (0.317–27.759)		
	Non-Veg	Group A 1 (50%)	0.600 (0.600–0.600)	0.0 (1.0)	2.121
		Group B 1 (50%)	1.672 (1.672–1.672)		
	Veg	Group A 20 (50%)	3.385 (0.317–7.720)	208.0 (.839)	0.864
		Group B 20 (50%)	2.710 (0.635–24.749)		
Stress Score	High	Group A 13 (59.09%)	4.070 (0.334–8.770)	63.0 (.789)	1.232
		Group B 9 (40.91%)	2.260 (0.653–24.749)		
	Low	Group A 17 (50%)	2.220 (0.317–7.720)	153.5 (.769)	0.851
		Group B 17 (50%)	1.672 (0.317–12.374)		
	Moderate	Group A 53 (48.18%)	2.341 (0.317–11.570)	1271.0 (.153)	0.953
		Group B 57 (51.82%)	3.170 (0.317–27.759)		
Exercise Scale	High	Group A 8 (65.54%)	2.380 (0.334–11.570)	25.0 (.524)	1.259
		Group B 5 (38.46%)	0.768 (0.635–7.692)		
	Low	Group A 30 (42.86%)	2.281 (0.317–7.720)	485.5 (.176)	0.822
		Group B 40 (57.14%)	3.339 (0.334–27.759)		
	Medium	Group A 19 (67.86%)	2.460 (0.317–8.770)	77.0 (.694)	1.845
		Group B 9 (32.14%)-	3.310 (0.317–23.070)		
	Sedentary	Group A 26 (47.27%)	2.013 (0.317–8.361)	413.0 (.549)	0.716
		Group B 29 (52.70%)	1.334 (0.317–18.300)		
Family History	Father	Group A 14 (41.18%)	2.0060 (0.334–7.360)	111.0 (.318)	0.804
		Group B 20 (58.82%)	3.587 (0.317–18.300)		
	Mat grandfather only	Group A 3 (75%)	0.950 (0.635–2.020)	2.5 (.637)	2.236
		Group B 1 (25%)	0.635 (0.635–0.635)		
	Mat grandmother only	Group A 6 (85.71%)	4.005 (0.600–8.770)	3.5 (1.0)	3.874
		Group B 1 (14.29%)	3.490 (3.490–3.490)		
	Mother	Group A 13 (46.43%)	2.860 (0.668–7.720)	84.0 (.549)	0.909
		Group B 15 (53.57%)	3.344 (0.334–24.749)		
	No history	Group A 39 (50.65%)	2.420 (0.317–11.570)	790.5 (.618)	0.848
		Group B 38 (49.35%)	2.195 (0.334–27.759)		
	Pat grandfather only	Group A 4 (66.67%)	3.965 (0.317–7.010)	4.0 (1.0)	1.889
		Group B 2 (33.33%)	3.500 (3.490–3.510)		
	Pat grandmother only	Group A 4 (40%)	1.955 (0.432–4.760)	7.0 (.352)	1.011
		Group B 6 (60%)	4.348 (0.668–12.709)		

## Discussion

Regular monitoring of DM is vital for early diagnosis, assessing the patient’s compliance, and evaluating if the anti-diabetic medication is working effectively. Blood is the most commonly used biological fluid to check the glycemic load. Many patients do not go for regular monitoring and blood glucose investigations because of the fear of blood withdrawal and needle pricking. Recently, saliva has gained popularity as a potential biological fluid for estimating blood glucose levels [18]. Saliva contains 3 to 25 mmol/l of glucose, which can be correlated with blood glucose levels. Many studies have correlated salivary and glucose levels in patients and shown that salivary fasting glucose levels in patients with DM have a statistically positive correlation to blood glucose levels in diabetic patients as compared to healthy controls [24–33,41–46]. However, a study by Amer et al. did not find any glucose, even in the slightest concentrations in the saliva of healthy individuals. However, the salivary samples obtained from Type 2 DM showed a significant concentration of glucose [19]. These findings are similar to the results obtained from our study, where a statistically significant difference in the distribution of blood glucose levels between diabetic and healthy patients was noted (*p*-value< .05), but the same could not be represented in the salivary glucose levels. We also noted a weak correlation of 0.4 between the blood glucose and salivary glucose levels for Group B (Diabetic) considering the significance level of *p* ≤ .05 [19]. The level of salivary glucose does not vary with gender, and the concentration of salivary glucose was not dependent on blood glucose [36]. Although a positive correlation exists between salivary and blood glucose levels in diabetic patients unlike, healthy controls. The varying results between salivary and blood glucose levels are attributed to the fact that salivary and blood glucose are dependent upon many environmental and patient-related factors. Hence if salivary glucose has to be correlated to blood glucose levels, the influence of these variables should be taken into consideration [33–42].

To our knowledge, few studies have assessed the effect of socioeconomic status, stress, physical activity, and diet on the salivary, salivary flow rate, and blood glucose levels [35–42]. We found that factors like age, alcohol consumption, monthly wages (financial status), and socioeconomic status are associated with blood glucose levels. However, the effect of these on salivary glucose is not statistically significant [7]. Patients above 40 years of age showed a statistically significant difference (with a large effect) in the salivary glucose levels for those who are healthy (median= 1.337) vs. patients with DM (median= 3.010). Diet influenced the correlation between salivary and blood glucose levels. For example, if saliva is used to check the glucose level immediately after a meal, higher values may be obtained. This is also noted by previous studies where the correlation between the glucose concentration in saliva and serum was higher after than before the carbohydrate intake. The results were also noted to be independent of factors such as age, gender, and duration of the disease [17,30,34]. The poor correlation between blood and salivary glucose levels in diabetic patients may also linked to the oral retention of carbohydrates, the flow of saliva, glucose utilization by bacteria, the release of carbohydrates from salivary glycoproteins, and contamination of the saliva by gingival crevicular fluid, especially in patients with poor periodontal status [35–38]. Jurysta et al. (2009) evaluated the salivary glucose concentration in unstimulated and mechanically stimulated salivary samples in normal, healthy, and diabetic patients and observed a higher glucose concentration in the saliva of diabetic patients than in healthy controls. Furthermore, no significant difference between unstimulated and stimulated salivary samples when compared with the serum glucose levels in diabetic patients was noted [39].

Based on the existing evidence and efficacy of saliva for blood glucose estimation, many companies have developed kits to test blood glucose using saliva. However, one should note that the sensitivity of these kits may be influenced by factors such as diet, stress, age, and stress levels in individuals. If future salivary kits are to be developed, one should know how patient-related factors such as age, BMI, socioeconomic status, stress, diet, and time of collection of saliva are matched to provide a more objective estimation of blood glucose using saliva. Age, socioeconomic status, and diet (especially consumption of fresh fruits and vegetables, stress, and monthly wages) influence salivary and blood glucose levels. One should also note that calibrating such devices with variables like monthly wages, stress, and diet would be a very difficult task as these variables are very dynamic and sometimes cannot be measured objectively. Hence some margins of error would always remain when saliva is used as a tool for measuring blood glucose. Additionally, one should note that the potential use of saliva in diagnosis as well as in the regular monitoring of diabetic patients cannot be attempted in certain situations including numerous auto-immune and/or inflammatory conditions such as Sjogren’s syndrome, amyloidosis, sarcoidosis, HIV/AIDS, hepatitis C, malignant conditions such as lymphomas and salivary gland agenesis or aplasia. Additionally, the use of saliva as a diagnostic tool is limited in patients with xerostomia or a possible change in salivary composition to the extent of not being reliable for diagnostics as well as in the regular monitoring of the patients. Patients with salivary gland changes after exposure to radiation in the head and neck area cancer also pose such challenges. Similar challenges are faced even in situations wherein the glucose threshold is either exceeded as in hyperglycaemic crises like diabetic ketoacidosis due to xerostomia or in cases of severe hypoglycaemia because blood glucose levels have to cross a minimum threshold to appear in saliva [40–46].

However, for healthy and diabetic individuals with good saliva flow rates, saliva can be used as a potential diagnostic tool for blood glucose estimation. Salivary glucose can be correlated to blood glucose levels and this correlation between saliva and blood glucose levels was found to be significant, but based on many factors. Any model or machine analyzing glucose levels using saliva should be calibrated based on these factors. Since the prevalence of DM is rising at a massive rate, the use of a non-invasive method of estimating blood glucose levels is crucial.

## Conclusion

Saliva is a potential biological fluid that can be used for glucose estimation. However, a weak correlation between saliva and blood glucose levels in healthy individuals was seen. However, salivary glucose was correlated to serum glucose for individuals with DM. Hence, saliva should only be used as a monitoring tool rather than a diagnostic tool, and that too only in highly diabetic patients.

## Supplementary Material

Correlational analysis between salivary and blood glucose levels in individuals with and without diabetes mellitus: a cross-sectional study

## Data Availability

Data related to the study is available upon request from *via* email to the corresponding author.

## Geolocation information

India
