# Supplementary material for: Correlational analysis between salivary and blood glucose levels in individuals with and without diabetes mellitus: a cross-sectional study
Source: Acta Odontol Scand. 2024 Mar 26;83:40293. doi: 10.1080/00016357.2023.2267678 (PMC11302642; doi:10.1080/00016357.2023.2267678)
Supplement: Correlational analysis between salivary and blood glucose levels in individuals with and without diabetes mellitus: a cross-sectional study [file AOS-83-40293-s1.pdf]

Supplementary material has been published as submitted. It has not been copyedited or typeset by Acta Odontologica Scandinavica.

Supplementary table 1: Analysis of Diet questionnaire with blood and salivary glucose

Question	Question Category	BG Level	Salivary Glucose Median [Min-Max]	Mann-Whitney U Test p-value
**Q1 (How often do you drink alcohol)**	1 - 2 times a day	Group A 1	0.334 (0.334-0.334)	0.637
		Group B 3	0.635 (0.334-0.669)	
	2 - 4 times a week	Group A 2	8.070 (7.370-8.770)	0.139
		Group B 3	4.0130 (3.010-4.013)	
	5 - 6 times a week	Group A 0	0	0.134
		Group B 1	4.0130 (4.0130-4.0130)	
	Never or very rarely	Group A 56	2.440 (0.317-8.361)	0.154
		Group B 58	3.240 (0.334-27.759)	
	Once a week or less often	Group A 22	2.273 (0.635-11.570)	0.669
		Group B 13	2.0735 (0.317-9.698)	
**Q2 (How often do you eat a portion of vegetables)**	1 - 2 times a day	Group A 37	2.540 (0.317-11.570)	0.650
		Group B 21	2.260 (0.334-27.759)	
	2 - 4 times a week	Group A 18	3.810 (0.644-8.770)	0.575
		Group B 15	2.860 (0.317-14.046)	
	3 or more times day	Group A 11	0.668 (0.317-7.370)	**0.009**
		Group B 30	3.252 (0.334-24.749)	
	5 - 6 times a week	Group A 11	2.460 (1.587-7.720)	0.647
		Group B 10	2.850 (0.635-17.056)	
	Never or very rarely	Group A 2	0.874 (0.796-0.952)	0.667
		Group B 1	0.317 (0.317-0.317)	
	Once a week or less often	Group A 2	4.460 (3.310-5.610)	1.0
		Group B 1	3.310 (3.310-3.310)	
**Q3 (Include fresh, tinned and frozen vegetables and pulses like**	1 - 2 times a day	Group A 28	2.113 (0.317-8.361)	**0.036**
		Group B 30	3.8455 (0.635-27.759)	
	2 - 4 times a week	Group A 27	2.54 (0.644-8.770)	**0.018**

**lentils and kidney beans)**		Group B<br>10	1.1125<br>(0.334-4.760)	
	3 or more times day	Group A<br>6	0.6680<br>(0.334-2.540)	**0.039**
		Group B<br>10	4.0130<br>(0.334-24.749)	
	5 - 6 times a week	Group A<br>7	2.8600<br>(0.668-7.720)	0.389
		Group B<br>11	3.344<br>(0.668-17.056)	
	Never or very rarely	Group A<br>6	3.0650<br>(0.635-5.710)	0.143
		Group B<br>5	0.7680<br>(0.317-2.17)	
	Once a week or less often	Group A<br>7	3.4900<br>(0.952-11.570)	0.352
		Group B<br>12	2.56<br>(0.317-10.870)	
**Q4<br>(How often did you eat a portion of fruit? Include fresh, frozen, tinned and dried fruit. Do not count fruit juices.)**	1 - 2 times a day	Group A<br>18	2.2200<br>(0.317-7.720)	0.892
		Group B<br>22	1.7850<br>(0.334-18.300)	
	2 - 4 times a week	Group A<br>23	2.860<br>(0.317-11.570)	0.144
		Group B<br>23	3.678<br>(0.635-24.749)	
	3 or more times day	Group A<br>0	0	
		Group B<br>2	4.3470<br>(0.668-8.026)	
	5 - 6 times a week	Group A<br>14	2.460<br>(0.432-7.720)	**0.048**
		Group B<br>14	3.7515<br>(1.003-27.759)	
	Never or very rarely	Group A<br>3	3.81<br>(0.952-5.710)	0.226
		Group B<br>4	1.0660<br>(0.317-2.86)	
	Once a week or less often	Group A<br>23	2.5400<br>(0.317-8.361)	0.531
		Group B<br>13	2.1700<br>(0.317-4.760)	
**Q5<br>(How often did you eat a cake, a sweet pastry like a Danish pastry, a donut or a sweet biscuit?)**	1 - 2 times a day	Group A<br>3	2.0060<br>(0.334-2.860)	0.629
		Group B<br>4	2.9710<br>(1.003-9.364)	
	2 - 4 times a week	Group A<br>16	3.4000<br>(0.432-8.770)	0.677
		Group B<br>14	2.240<br>(0.635-24.749)	
	5 - 6 times a week	Group A<br>6	2.6075<br>(0.950--7.360)	0.931
		Group B<br>5	3.160<br>(0.669-27.759)	
	Never or very rarely	Group A<br>13	4.3800<br>(0.334-11.570)	0.464
		Group B<br>17	2.0735<br>(0.334-18.300)	
		Group A	1.9000	0.124

	Once a week or less often	43	(0.317-7.720)	
		Group B 38	3.334 (0.317-17.056)	
**Q6 (How often did you eat sweets, chocolate or sugary foods like gulab jamun, halva or sweet popcorn?)**	1 - 2 times a day	Group A 3	1.370 (0.334-2.341)	1.0
		Group B 1	1.003 (1.003-1.003)	
	2 - 4 times a week	Group A 20	2.9925 (0.635-7.720)	0.124
		Group B 12	6.1810 (0.635-17.056)	
	5 - 6 times a week	Group A 7	5.7100 (3.17-7.360)	0.074
		Group B 5	3.3100 (2.17-4.76)	
	Never or very rarely	Group A 12	2.1130 (0.334-8.361)	0.768
		Group B 23	2.0735 (0.334-24.749)	
	Once a week or less often	Group A 39	1.900 (0.317-11.570)	0.182
		Group B 37	3.3340 (0.317-27.759)	
**Q7 (How often did you drink sugary drinks? Include non-diet fizzy drinks, squashes, mixers, energy drinks, fruit juices, sweetened milk drinks or coffee, tea or other hot drinks with sugar or flavoured syrups.)**	1 - 2 times a day	Group A 14	2.500 (0.317-7.720)	1.0
		Group B 12	2.1950 (0.317-23.070)	
	2 - 4 times a week	Group A 25	2.5400 (0.334-7.720)	0.948
		Group B 12	3.4120 (0.317-10.870)	
	3 or more times day	Group A 2	3.8100 (2.86-4.760)	0.414
		Group B 2	1.7475 (0.635-2.860)	
	5 - 6 times a week	Group A 7	3.1700 (2.22-8.770)	0.152
		Group B 6	2.26 (0.635-4.760)	
	Never or very rarely	Group A 10	2.113 (0.334-11.57)	0.289
		Group B 24	3.257 (0.334-24.749)	
	Once a week or less often	Group A 23	2.0060 (0.317-8.361)	0.176
		Group B 22	3.3390 (0.334-27.759)	
**Q8 (How often did you use butter, full-fat margarine, ghee, lard or coconut oil or palm oil on your bread, potatoes or vegetables or in cooking?)**	1 - 2 times a day	Group A 38	1.785 (0.317-11.570)	0.073
		Group B 42	3.085 (0.334-18.300)	
	2 - 4 times a week	Group A 12	3.660 (0.668-5.710)	0.509
		Group B 10	3.40 (0.317-7.692)	
	3 or more times day	Group A 3	2.020 (0.950-7.720)	0.381
		Group B 6	5.852 (1.350-23.070)	

	5 - 6 times a week	Group A<br>11	4.38<br>(1.338-8.770)	0.805
		Group B<br>10	3.1365<br>(0.334-24.749)	
	Never or very rarely	Group A<br>5	3.81<br>(2.220-7.37)	0.286
		Group B<br>4	1.62775<br>(0.669-8.770)	
	Once a week or less often	Group A<br>12	2.113<br>(0.317-5.710)	0.851
		Group B<br>6	2.5150<br>(0.317-27.759)	
**Q9<br>(How often did you eat oily fish? Think about fresh or tinned salmon, trout, sardine, mackerel, pilchards, herring, red mullet, or fresh tuna.)**	1 - 2 times a day	Group A<br>1	0.334<br>(0.334-0.334)	0.667
		Group B<br>2	6.672<br>(0.635-12.709)	
	2 - 4 times a week	Group A<br>15	1.587<br>(0.334-4.380)	0.129
		Group B<br>22	2.38<br>(0.334-17.056)	
	5 - 6 times a week	Group A<br>3	3.17<br>(0.668-6.670)	0.786
		Group B<br>5	4.682<br>(0.334-14.046)	
	Never or very rarely	Group A<br>45	2.860<br>(0.317-11.570)	0.521
		Group B<br>29	2.860<br>(0.317-24.749)	
	Once a week or less often	Group A<br>17	2.540<br>(0.334-8.361)	0.368
		Group B<br>20	3.322<br>(0.334-27.759)	

Supplementary Table 2: Analysis of stress with blood and salivary glucose

Question	Question Category	BG Level	Salivary Glucose Median [Min-Max]	Mann-Whitney U Test p-value
**Q1 (How often have you been upset because of something that happened unexpectedly?)**	0 - Never	Group A 4	0.794 (0.317-1.670)	0.212
		Group B 3	2.074 (0.875-4.670)	
	1 - Almost Never	Group A 12	4.095 (0.668-7.720)	0.316
		Group B 5	0.334 (0.317-14.046)	
	2 - Sometimes	Group A 35	2.220 (0.317-11.570)	0.151
		Group B 39	3.170 (0.334-18.300)	
	3 - Fairly Often	Group A 20	4.095 (0.334-8.361)	0.909
		Group B 22	3.490 (0.317-27.759)	
	4 - Very Often	Group A 8	2.733 (0.334-8.770)	0.799
		Group B 4	3.512 (0.668-9.364)	
**Q2 (How often have you felt that you were unable to control the important things in your life?)**	0 - Never	Group A 8	2.120 (0.317-7.720)	0.921
		Group B 3	0.8750 (0.334-11.37)	
	1 - Almost Never	Group A 13	1.9000 (0.635-7.720)	0.283
		Group B 11	0.6690 (0.317-17.056)	
	2 - Sometimes	Group A 26	2.4405 (0.317-11.570)	**0.042**
		Group B 30	3.4900 (0.334-18.300)	
	3 - Fairly Often	Group A 20	2.660 (0.334-8.361)	0.791
		Group B 22	2.5600 (0.317-27.759)	
	4 - Very Often	Group A 12	3.660 (0.317-8.770)	0.554
		Group B 7	3.678 (1.003-10.870)	
**Q3 (How often have you felt nervous and stressed?)**	0 - Never	Group A 3	1.670 (0.644-5.610)	1.0
		Group B 3	0.6690 (0.669-11.370)	
	1 - Almost Never	Group A 14	1.645 (0.635-11.570)	0.965
		Group B 15	3.160 (0.317-14.046)	
	2 - Sometimes	Group A	2.341	0.545

		33	(0.317-7.720)	
		Group B 25	2.540 (0.334-17.056)	
	3 - Fairly Often	Group A 15	3.310 (0.317-7.720)	0.641
		Group B 20	3.417 (0.317-0.317)	
	4 - Very Often	Group A 14	3.940 (0.334-8.770)	0.266
		Group B 10	4.3865 (0.668-27.759)	
**Q4 (How often have you felt confident about your ability to handle your personal problems?)**	0 - Never	Group A 0	0	
		Group B 1	3.678 (3.678-3.678)	
	1 - Almost Never	Group A 7	1.338 (0.334-7.720)	0.182
		Group B 8	3.252 (0.950-24.749)	
	2 - Sometimes	Group A 21	3.170 (0.432-8.361)	0.378
		Group B 21	4.0130 (0.317-27.759)	
	3 - Fairly Often	Group A 32	2.2805 (0.317-11.570)	0.389
		Group B 23	3.3100 (0.334-23.070)	
	4 - Very Often	Group A 19	2.5400 (0.635-8.770)	0.603
		Group B 20	2.215 (0.317-14.046)	
**Q5 (How often have you felt that things were going your way?)**	0 - Never	Group A 2	6.535 (5.710-7.360)	0.667
		Group B 1	9.364 (9.364-9.364)	
	1 - Almost Never	Group A 12	2.280 (0.334-5.016)	0.241
		Group B 11	3.310 (0.334-24.749)	
	2 - Sometimes	Group A 34	2.460 (0.317-11.570)	0.232
		Group B 31	3.344 (0.317-17.056)	
	3 - Fairly Often	Group A 26	2.508 (0.317-8.770)	0.724
		Group B 21	3.010 (0.334-27.759)	
	4 - Very Often	Group A 5	0.796 (0.668-1.670)	0.789
		Group B 9	1.003 (0.317-11.370)	
**Q6 (How often have you found that you could not cope with all the things that**	0 - Never	Group A 4	2.740 (1.20-5.610)	0.686
		Group B 4	2.093 (0.317-11.370)	
	1 - Almost Never	Group A 15	1.900 (0.317-11.5700	0.743
		Group B 14	2.106 (0.334-12.709)	

**you had to do?)**	2 - Sometimes	Group A 32	2.220 (0.334-7.010)	0.078
		Group B 28	3.344 (0.334-23.07)	
	3 - Fairly Often	Group A 21	3.31 (0.317-7.360)	0.220
		Group B 20	3.846 (0.317-27.759)	
	4 - Very Often	Group A 7	5.71 (0.334-8.770)	0.337
		Group B 7	2.170 (0.334-24.749)	
**Q7 (How often have you been able to control irritations in your life?)**	0 - Never	Group A 3	1.587 (0.334-3.510)	0.857
		Group B 4	2.1735 (0.317-4.013)	
	1 - Almost Never	Group A 7	2.006 (0.635-2.860)	0.130
		Group B 10	3.252 (0.668-24.749)	
	2 - Sometimes	Group A 27	3.31 (0.317-8.77)	0.248
		Group B 25	3.510 (0.317-27.759)	
	3 - Fairly Often	Group A 31	2.460 (0.317-11.570)	0.829
		Group B 26	2.775 (0.334-23.070)	
	4 - Very Often	Group A 11	2.22 (0.668-7.720)	0.589
		Group B 8	0.8855 (0.668-14.046)	
**Q8 (How often have you felt that you were on top of things?)**	0 - Never	Group A 6	3.810 (1.200-5.710)	0.867
		Group B 2	2.8365 (1.003-4.670)	
	1 - Almost Never	Group A 14	1.7435 (0.334-4.440)	**0.041**
		Group B 9	3.6780 (0.668-27.759)	
	2 - Sometimes	Group A 27	3.5100 (0.317-11.57)	0.797
		Group B 31	3.3340 (0.334-24.749)	
	3 - Fairly Often	Group A 26	2.1130 (0.317-7.370)	0.288
		Group B 18	3.412 (0.317-23.070)	
	4 - Very Often	Group A 6	2.1725 (0.668-7.720)	0.758
		Group B 13	1.6720 (0.317-12.374)	
**Q9 (How often have you been angered because of things that**	0 - Never	Group A 3	3.810 (2.020-5.610)	0.8
		Group B 2	2.6525 (0.635-4.670)	
	1 - Almost Never	Group A 17	2.675 (0.317-7.720)	0.492
		Group B	3.327	

**happened that were outside of your control?)**		12	(0.334-14.046)	
	2 - Sometimes	Group A 36	1.953 (0.317-7.720)	0.453
		Group B 26	2.260 (0.334-23.070)	
	3 - Fairly Often	Group A 21	3.310 (0.334-11.570)	0.269
		Group B 24	4.0130 (0.317-27.759)	
	4 - Very Often	Group A 2	4.552 (0.334-8.770)	0.909
		Group B 9	2.170 (0.317-17.056)	
**Q10 (How often have you felt difficulties were piling up so high that you could not overcome them?)**	0 - Never	Group A 4	1.945 (0.668-5.610)	0.8
		Group B 2	6.1225 (0.875-11.370)	
	1 - Almost Never	Group A 18	2.500 (1.270-7.720)	0.952
		Group B 13	3.1700 (0.317-14.046)	
	2 - Sometimes	Group A 38	2.0130 (0.317-11.570)	0.436
		Group B 33	3.010 (0.317-24.749)	
	3 - Fairly Often	Group A 13	3.46 (0.334-7.36)	0.321
		Group B 21	3.6780 (0.668-18.30)	
	4 - Very Often	Group A 6	5.895 (1.338-8.77)	1.0
		Group B 3	2.260 (1.340-17.056)	
	Once a week or less often	Group A 0	0	
		Group B 1	27.7590 (27.7590-27.7590)	
